# Aspirin/amoxicillin loaded chitosan microparticles and polydopamine modified titanium implants to combat infections and promote osteogenesis

**DOI:** 10.1038/s41598-024-57156-1

**Published:** 2024-04-01

**Authors:** Yun Shi, Yongzhen Lai, Yan Guo, Zhiyu Cai, Chuanqing Mao, Meng Lu, Chengyan Ren, Joo L. Ong, Weihui Chen

**Affiliations:** 1 Department of Oral and Maxillofacial Surgery, Fujian Medical University Union Hospital, Fuzhou, Fujian China; 2 Fujian Key Laboratory of Oral Diseases & Fujian Provincial Engineering Research Center of Oral Biomaterial & Stomatological Key Lab of Fujian College and University, School and Hospital of Stomatology, Fujian Medical University, Fuzhou, China; 3 Department of Biomedical Engineering and Chemical Engineering, University of Texas at San Antonio, One UTSA Circle, San Antonio, TX 78249 USA

**Keywords:** Aspirin, Amoxicillin, Antibacterial, Osteogenesis, Dental implants, Biomaterials, Microbiology

## Abstract

It is known that titanium (Ti) implant surfaces exhibit poor antibacterial properties and osteogenesis. In this study, chitosan particles loaded with aspirin, amoxicillin or aspirin + amoxicillin were synthesized and coated onto implant surfaces. In addition to analysing the surface characteristics of the modified Ti surfaces, the effects of the modified Ti surfaces on the adhesion and viability of rat bone marrow-derived stem cells (rBMSCs) were evaluated. The metabolic activities of *Staphylococcus aureus* (*S. aureus*) and *Escherichia coli* (*E. coli*) biofilms on the modified Ti surfaces were also measured in vitro. Moreover, *S. aureus* was tested for its antibacterial effect by coating it in vivo. Using water as the droplet medium, the contact angles of the modified Ti surfaces increased from 44.12 ± 1.75° to 58.37 ± 4.15°. In comparison to those of the other groups tested, significant increases in rBMSC adhesion and proliferation were observed in the presence of aspirin + amoxicillin-loaded microspheres, whereas a significant reduction in the metabolic level of biofilms was observed in the presence of aspirin + amoxicillin-loaded microspheres both in vitro and in vivo. Aspirin and amoxicillin could be used in combination to coat implant surfaces to mitigate bacterial activities and promote osteogenesis.

## Introduction

Since the 1970s, there have been numerous efforts to advance dental implantology, and the use of dental implants has since become an indispensable part of clinical dentistry^1^. Although a 10-year prospective multicentre study indicated a greater than 90% survival rate for dental implants^2^, a rapid increase in the number of elderly people worldwide^3^ may alter the high survival rate, as ageing is related to an increase in tooth loss as well as other health-related factors. Diabetes, osteoporosis, obesity and drug use are all health-related issues that may play a role in the overall health of the bone around dental implants and possibly impact implant fixation and implant success^1^. Although titanium (Ti) and Ti alloys are known to have excellent mechanical properties and biocompatibility, they are not known to be osteoconductive or osteo-inductive and therefore do not promote early osseointegration. In addition, bacterial adhesion and biofilm formation on the Ti implant surface are still the most common causes of implant infection in many patients^4^. As a result, one of the objectives of clinical implantology is to improve implant success by developing and investigating novel implant surfaces that dually promote osteogenesis and antibacterial activity.

It is known that both subtractive and additive surface modifications of Ti implant surfaces have greatly improved implant fixation and soft tissue adhesion to implants^5^. Subtractive modifications include sandblasting, which results in surface roughening^5^, whereas additive modifications include layering down coatings to release antibacterial drugs or osteo-inductive molecules^6,7^. At present, most surface modifications tend to either promote osseointegration or enhance the antibacterial properties of the material. In recent years, physical, chemical, electrochemical and other modification methods have been employed to promote dual osseointegration and resist bacterial adhesion^8–13^, but most of these modification techniques are complicated and/or equipment intensive and are difficult to translate in clinical practice.

In addition to modification techniques, responses to implant surfaces are also highly dependent on biomaterial selection. The use of chitosan microspheres has attracted much attention due to their nontoxicity, biocompatibility and biodegradability. As polycations that can complex with various anions, chitosan is also an excellent carrier and thus is often used to encapsulate biomolecules^14–17^.

Like in the selection of biomaterials, the choice of drugs used for encapsulation is also highly dependent on the application of the material. Aspirin (ASA) is a classic nonsteroidal anti-inflammatory drug that is known for its antipyretic, analgesic and anti-inflammatory effects. As an excellent bone-enhancing drug, ASA has recently been reported to promote osteogenesis by inhibiting osteoclast differentiation, activating osteoblasts, promoting osteogenic differentiation of bone marrow mesenchymal stem cells, reducing bone resorption and improving bone regeneration^18–26^.

Like in any surgery, the wound site is prone to bacterial infection. Although systemic application of antibiotics is enough to reduce the risk of early implantation failure in healthy patients^27^, there may be corresponding side effects such as drug resistance caused by excessive use^28^, damage to systemic organs and allergic reactions. Unlike in the systemic delivery of antibiotics, the application of antibiotics directly or locally to the wound site from implant coatings^7,29,30^ has been reported to accurately and appropriately achieve antibacterial effects as well as reduce implant failure^31^. One example of an antibiotic that is often used for systemic prophylactic medication to improve implant success in oral implant surgery is amoxicillin (AMO). Studies on the perioperative systemic use of amoxicillin in implant surgery have shown that AMO may be good enough to reduce the failure rate of early dental implants to 2%. However, the special antibiotic regimen may be effective due to the excessive use of antibiotics and systemic side effects^32,33^. Local and low-dose application of antibiotics could achieve moderate and effective antibacterial effects^34^. Adding amoxicillin to the implant surface may achieve a good antibacterial effect.

Therefore, the objectives of this study were to (1) characterize Ti surfaces coated with aspirin- and/or amoxicillin-loaded chitosan particles; (2) evaluate the effect of modified Ti surfaces on the adhesion and viability of rat bone marrow-derived stem cells (rBMSCs); (3) measure the metabolic activity of *Staphylococcus aureus* (*S. aureus*) and *Escherichia coli* (*E. coli*) biofilms on modified Ti surfaces in vitro; and (4) measure the metabolic activity of *S. aureus* biofilms on modified Ti surfaces in vivo.

## Materials and methods

### Preparation and morphological analysis of drug loaded chitosan microparticles (CS-MPs)

Drug-loaded chitosan microparticles were prepared using the double emulsion-crosslinking method. Based on the known synthesis system^35^, we synthesized microspheres with different doses of aspirin and amoxicillin. According to the stability of the synthesis system and the morphology of the microspheres, 40 mg of aspirin and 20 mg of amoxicillin were ultimately selected for microsphere synthesis. Briefly, an internal oil in water (O/W) emulsion was formed with 2% (w/v) chitosan acetic acid solution (6 mL) as the aqueous phase and 40 mg of aspirin (A2093, Sigma‒Aldrich, Shanghai, China) and/or 20 mg of amoxicillin (A822839, Macklin, Shanghai, China) in methylene chloride as the oil phase. Subsequently, an external W/O emulsion with liquid paraffin as the oil phase, span-80 as the emulsifier and glutaraldehyde as the aqueous phase crosslinking agent was also prepared. Next, the external W/O emulsion was dropped into the internal O/W emulsion, and the obtained mixture was then stirred for 2 h to synthesize crosslinked drug-loaded chitosan microparticles (CS-MPs).

#### Particle size and morphology

The morphology of the drug-loaded CS-MPs (CS-ASA MPs, CS-AMO MPs, and CS-ASA/AMO MPs) was characterized using a scanning electron microscope (SEM) (Gemini 500, ZEISS, Germany) and transmission electron microscopy (TEM) (Tecnai G2 F20, FEI, USA) at ×50 K, ×70 K and ×2 K magnification. The particle size and dispersity were determined via dynamic light scattering (DLS) using a Zeta Sizer (ZS90, Malvern, UK) at a temperature of 25 °C. With a sample size (n) of 3 per group, particle size measurements were carried out by suspending the CS-MPs in deionized water. Each sample was measured in triplicate.

#### Molecular composition

Using a sample size (n) of 3 per group, the functional groups of chitosan, ASA, AMO and their derivatives were determined using Fourier transform infrared (FT-IR) spectroscopy (ALPHAII, Bruker, Germany). The sample was prepared into a thin slice. Spectra were recorded from 4000 to 500 cm^−1^ by the attenuated total reflection (ATR) method. By identifying the functional groups with characteristic vibration frequencies in the infrared region, the chemical and structural information of the samples was obtained.

#### Encapsulation efficiency

Briefly, ASA and/or AMO were extracted from the CS-MPs using phosphoric acid and acetonitrile via an ultrasonic-assisted method. The same amount of phosphoric acid (30417, Sigma‒Aldrich, Shanghai, China) and acetonitrile (1.00030, Merck, Germany) were added to a quarter of each sample, and the particles were subsequently dissolved after ultrasonic treatment for 30 min. After ultrasonic treatment, the mixture was filtered through a 0.22 μm filter membrane, after which the drug concentration was detected. The supernatant solution containing ASA and/or AMO was subsequently measured via liquid chromatography (1260 Infinity II Prime LC, Agilent, USA) at a wavelength of 228 nm for ASA or 230 nm for AMO. The drug encapsulation efficiency was then calculated using the following expression (Eq. (1)):

1 $$Encapsulation\,efficiency \left(\%\right)=\frac{Weight\,of\,drug\,in\,CS-MPs}{Weight\,of\,drug\,used\,during\,preparation}\times 100$$

#### Drug release kinetics

Briefly, 40 mg samples (CS-ASA MPs, CS-AMO MPs, and CS-ASA/AMO MPs) were separately placed in 100 mL of potassium phosphate buffer (pH 7.4) and maintained at 37 °C with a shaking speed of 180 rpm. One millilitre of dialysate was collected every 24 h from the released medium for each sample, after which the mixture was replaced with 1 mL of fresh potassium phosphate buffer. The absorbance was read using liquid chromatography (1260 Infinity II Prime LC, Agilent, USA) at a wavelength of 228 nm for ASA and 230 nm for AMO. The drug release ratio was then expressed as a percentage of the total drug available and plotted as a function of time.

### Preparation and surface characterization of modified titanium (Ti) disks

#### Control Ti surfaces (SLA group)

Aluminium oxide was sprayed evenly and vertically on the surface of the titanium sheet, and the distance from the titanium sheet was set to 1 cm. After sandblasting, the titanium sheet was placed into a mixed etching solution with a V (H_2_O): V (H_2_SO_4_): V (HCl) ratio of 11:12:2 for 1 min. After acid etching, the sandblasted and acid-etched (SLA) Ti disks were ultrasonically cleaned with deionized water for 15 min and subsequently dried at room temperature. All the titanium sheets were disinfected with ethylene oxide for later use. These Ti-SLAs were subsequently used as a control group.

#### Coating on Ti-SLA surfaces (PDA group)

Polydopamine (PDA) coating was applied to Ti-SLA surfaces in an alkaline environment. PDA coatings can be used as intermediate coatings to modify the surface of titanium by grafting related drugs, proteins and ions. Two grams of dopamine hydrochloride (D806618; Macklin, China) was prepared in a dopamine solution with a concentration of 1 L of Tris-HCl (FD7981; FUDE Biological Technology, China) (pH 8.5). The cleaned Ti-SLA plates were soaked in dopamine solution, placed in a dark environment and reacted under normal oxygen conditions for 24 h. The polydopamine-coated SLA surfaces (Ti-PDA) were ultrasonically cleaned with deionized water for 30 min to remove the unattached PDA on the surface of the Ti-SLA plates.

#### Incorporation of drug-loaded CS-MPs on Ti-PDA coatings

First, 2 g of CS-ASA MPs, CS-AMO MPs or CS-ASA/AMO MPs were suspended in 1 L of deionized water for 6 h, after which the drug-loaded CS-MPs reacted and firmly bound the drug-loaded CS-MPs to the PDA coating. This reaction was achieved by reacting PDA with a Schiff base (C=N chemical bond), which can be formed by PDA and the amino group of chitosan in aqueous solution, thereby resulting in the following three drug-loaded CS-MP groups: (1) aspirin-loaded CS-MPs on PDA-coated SLA surfaces (aspirin group), (2) amoxicillin-loaded CS-MPs on PDA-coated SLA surfaces (amoxicillin group), and (3) aspirin + amoxicillin-loaded CS-MPs on PDA-coated SLA surfaces (aspirin + amoxicillin group).

#### Surface morphology and roughness measurement analyses

The surface morphology of the control and experimental groups was examined using SEM (EM 8000, KYKY Technology, China) at ×10K magnification. Three random spots per sample were scanned. In addition, the average roughness (Ra) of the surfaces at three random regions per sample was measured using an atomic force microscope (AFM) (Dimension Icon, Bruker, Germany). The samples were also analysed via Raman spectroscopy, and an excitation wavelength of 633 nm was used. The acquisition time was set to 10 s. Three randomly selected sites were analysed for each sample.

#### Molecular composition and contact angle measurements

The thickness of the Ti disks was not suitable for FT-IR detection, and the Raman spectrum was ultimately selected for detection. Using a sample size (n) of 3 per group, the functional groups on the Ti-PDA-coated surfaces before and after the incorporation of CS-MPs were determined via Raman spectroscopy (RM2000, Renishaw, UK). An excitation wavelength of 633 nm was used, and the beam intensity was approximately 10 mW. The acquisition time was set to 10 s. The spectra were recorded from 0 to 3500 cm^−1^. Three randomly selected sites were analysed for each sample. Static contact angle measurements were also performed using water as the droplet (SINDIN SDC 100, SHENGDING Precision Instrument, China). Using the sessile drop method in air, 5 μL deionized water droplets were placed on the surface of each sample (n = 6), and the contact angles were measured within 10 s.

### Adhesion and viability of rat bone marrow-derived stem cells (rBMSCs) on the surfaces

#### Cell isolation

For cell culture, rat bone marrow-derived stem cells (rBMSCs) were isolated from the bone marrow of 4-week-old Sprague–Dawley rats. The rBMSCs were cultured in Dulbecco's modified Eagle’s medium (DMEM) supplemented with low glucose (HyClone, USA) and 10% foetal bovine serum (FBS; Wisent, Canada) at 37 °C in a humidified atmosphere of 5% CO_2_. The DMEM was changed every 3 days during cell culture, and the experiments were carried out with the rBMSCs after 2–5 passages.

#### Adhesion and viability assay

A Cell Counting Kit-8 (CCK-8) (M4839, AbMole, China) was used to assess the viability of the rBMSCs seeded in all groups. Briefly, control and experimental Ti surfaces were placed in 24-well plates and seeded with 1 ×10^4^ rBMSCs/mL DMEM. All the samples were cultured at 37 °C in 5% CO_2_, and the media was changed every 3 days. After 1, 4, and 7 days, the specimens were removed from the 24-well plate and washed three times with phosphate-buffered saline (PBS) before being transferred to new 24-well plates. Then, 500 µl of media containing 10% CCK-8 solution was added to each well, which was followed by 2 h of incubation, after which the optical density (OD) of the media was read at 450 nm. After 3 days, the cells were stained with DAPI Staining Solution (Beyotime Biotechnology, China) and Actin-Tracker Red-Rhodamine (Beyotime Biotechnology, China) for cytoskeleton analysis via an inverted epifluorescence microscope (IX71, Olympus, Japan).

### In vitro evaluation of antibacterial effects

#### Metabolic activity of the biofilm formed

Gram-positive *Staphylococcus aureus* (ATCC25923) and gram-negative bacteria *Escherichia coli *(BNCC269342; BeNa Culture Collection, China) are common implant-related pathogens that can cause infections. These microbles were used to measure the metabolic activity of biofilms formed on control and modified Ti surfaces by SEM and colony counting methods. Briefly, *S. aureus* and *E. coli* were separately inoculated in Luria–Bertani broth (LB) at 37 °C. The bacterial concentration was adjusted to 1 ×10^6^ CFU/mL or 1 ×10^8^ CFU/mL, and the bacteria were incubated on control or modified Ti surfaces for 24 h. After 24 h of incubation, the biofilms that formed on the Ti surfaces were washed twice with PBS and transferred to new 24-well plates. The morphology of the bacteria on the surfaces of these samples was viewed by SEM after 2.5% C_3_H_5_(CHO)_2_ fixation, C_2_H_5_OH dewatering, and gold spraying. For colony counting, using a sample size of 6 per group, each fabricated sample was transferred to a tube containing PBS. After ultrasonic treatment for 15 min, the bacterial suspension was diluted according to the gradient and uniformly coated on an agar plate. The number of colony-forming units (CFUs) was measured after the cells were cultured in a temperature incubator (37 °C) for 24 h.

#### Live/dead bacterial staining

Using a sample size of 3 per group, each Ti disk was washed three times with PBS to remove nonadherent bacteria. The disks were then stained with a Bac Light live/dead bacterial viability kit (MX4234, MKbio, China), which contains 2 nucleic acid stains, a green fluorescent SYTO^™^9 stain for live bacteria and a red fluorescent propidium iodide stain for dead bacteria. At random locations on each disk, five images of live/dead bacteria were obtained using an Olympus IX71 inverted epifluorescence microscope (IX71, Olympus, Japan), resulting in 15 images per group.

### In vivo Implantation

#### Surgical implantation

Ten male Sprague–Dawley (SD) rats aged >8 weeks and with a body weight of 230 ± 26 g were included in this study. The procedures were approved by the Animal Ethics Committee (Approval No.: IACUC FJMU 2023-0081). Animals were anaesthetized with xylazine hydrochloride (Sigma, USA) at a dosage of 5 mg/kg and ketamine hydrochloride (Jiangsu Hengrui Medicine Co., Ltd., China) at a dosage of 50 mg/kg. Afterward, the dorsal area of each rat was shaved and disinfected, and five asymmetric midline incisions of the same size (1.5−2 cm) were created. Sharp and blunt dissection were used to develop subcutaneous pockets beneath the incisions. The fabricated samples (SLA group, PDA group, aspirin group, amoxicillin group, and aspirin + amoxicillin group) were subcutaneously implanted into five rats. A 100 μL injection of the bacterial suspension of *S. aureus* was injected into the implant position. The rats were euthanized by inhaling carbon dioxide after 24 h. This study was carried out in compliance with the ARRIVE guidelines, and all the experiments were carried out in strict accordance with the recommendations of the laboratory of Fujian Medical University.

#### Colony counting method

The implanted samples were removed to observe their antibiotic efficacy via the plate colony counting method. The implanted samples were soaked completely in saline, and the solution was used for bacterial quantification after ultrasound. After incubating at 37 °C for 24 h, the bacterial colonies were counted.

#### SEM

The surface morphologies of the control and experimental groups were examined using SEM (EM 8000, KYKY Technology, China) at ×3K magnification. Three random spots per sample were scanned.

#### Haematoxylin and eosin (HE) staining

After incubating at 37 °C for 24 h, the surrounding skin and tissue were collected for HE staining to observe their levels of inflammation. A 10% neutralized buffered formalin solution was used to fix the cells for 24 h, after which the standard method was used for processing.

### Statistical analysis

The quantitative data collected are expressed as the mean ± standard deviation (mean ± sd). To ensure repeatability, each in vitro study was performed three different times. With SPSS software (v19.0, IBM Corp., Armonk, NY, USA), statistical analysis of all the collected data was performed via one-way analysis of variance (ANOVA), and a statistically significant difference and a highly significant difference were considered at p < 0.05 and p < 0.01, respectively.

### Ethics approval and consent to participate

The procedures were approved by the Animal Ethics Committee of Fujian Medical University Union Hospital (Approval No.: IACUC FJMU 2023-0081). This study was carried out in compliance with the ARRIVE guidelines and all experiments were carried out in strict accordance with the recommendations in the laboratory of Fujian Medical University.

## Results

### Morphological analysis of the drug-loaded CS-MPs

**Figure 1:**
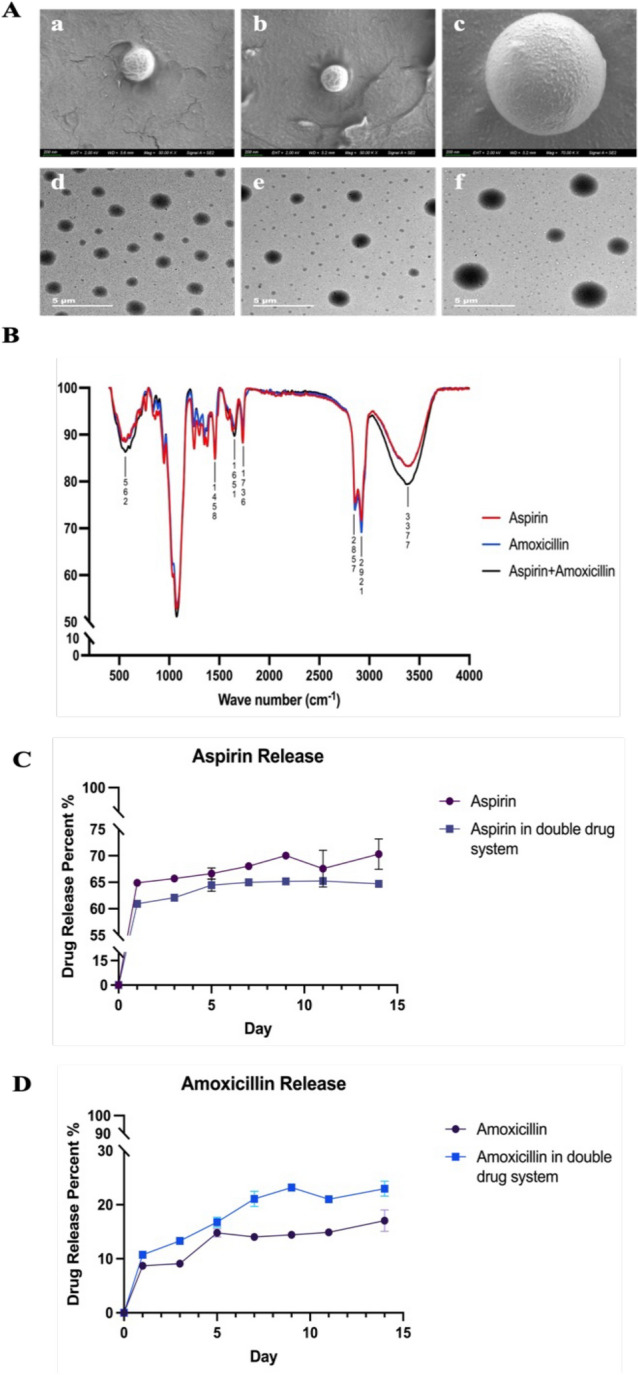
SEM images of (**A.a**) CS-ASA MPs, (**A.b**) CS-AMO MPs and (**A.c**) CS-ASA/AMO MPs showing the size of the drug-loaded chitosan particles at ×50 K and ×70 K. TEM images of (**A.d**) CS-ASA MPs, (**A.e**) CS-AMO MPs and (**A.f**) CS-ASA/AMO MPs showing the surface topography of the drug-loaded chitosan particles at ×2 K. FT-IR spectra of three kinds of drug-loaded chitosan microspheres (**B**). Drug sustained release percent diagram of (**C**) aspirin release in single drug-loaded particles and double drug-loaded particles and (**D**) amoxicillin release in single drug-loaded particles and double drug-loaded particles in potassium phosphate buffer (pH 7.4).

Using SEM and TEM, Fig. 1A shows the morphology of the chitosan drug-loaded microspheres among the three different drug-loaded microspheres investigated in this study. Table 1 shows that the diameter of the microspheres loaded with amoxicillin and aspirin was 1132.85 ± 30.93 nm. Table 1 also shows that the polydispersity index of the amoxicillin-loaded microspheres was 0.239 ± 0.02. Figure 1C shows that aspirin had a burst release on Day 1 at approximately 60–65% for both the CS-ASA MPs and the CS-ASA/AMO MPs, followed by a steady release over the other 13 days. However, amoxicillin caused mild release of approximately 10% (Fig. 1D) of both the CS-AMO MPs and the CS-ASA/AMO MPs on Day 1.

**Table 1:** Particle size and dispersity of three drug-loaded chitosan particles.

	CS-ASA MPs	CS-AMO MPs	CS-ASA/AMO MPs
Average diameter (nm)	314.09 ± 9.25	287.45 ± 15.8	1132.85 ± 30.93
Polydispersity index (PDI)	0.338 ± 0.04	0.239 ± 0.02	0.369 ± 0.01

*CS-ASA MPs, chitosan aspirin microparticles; CS-AMO MPs, chitosan amoxicillin microparticles; CS-ASA/AMO MPs, chitosan aspirin/amoxicillin microparticles.

Table 2 shows 40.8 ± 1.9% and 57.4 ± 12% drug encapsulation efficiency of aspirin and amoxicillin, respectively, in aspirin or amoxicillin drug-loaded chitosan microspheres, whereas the encapsulation efficiency in aspirin + amoxicillin drug-loaded microspheres decreased to 36.9 ± 2.5% and 34.6 ± 14.1% for aspirin and amoxicillin, respectively (p < 0.05).

**Table 2:** Drug encapsulation efficiency (EE).

	Single drug-loaded CS-MPs	Aspirin + amoxicillin drug-loaded CS-MPs
Aspirin EE (%)	40.84 ± 1.9	36.86 ± 2.5
Amoxicillin EE (%)	57.4 ± 12	34.58 ± 14.1

*CS MPs, chitosan microparticles.

As observed in Fig. 1B, the FT-IR characteristic peaks of aspirin (~1458 cm^−1^, ~1736 cm^−1^) and amoxicillin (~2857 cm^−1^, ~2921 cm^−1^, ~1736 cm^−1^) were visible in the spectra of the double drug-loaded microspheres. The distinct regions of the characteristic peaks of amoxicillin at 3000–2800 cm^−1^ ~2857, ~2921 cm^−1^ and ~1736 cm^−1^ correspond to the stretching modes of –CH_2_ and –CH_3_, respectively, of the various membrane amphiphiles and ester bands, whereas the characteristic peak of aspirin at ~1736 cm^−1^ corresponds to the stretching mode of –CH_3_ in the ester band. In addition, the ~1458 cm^−1^ peak for aspirin corresponded to the –COO– group. The FT-IR characteristic peaks for chitosan (~3377 cm^−1^ and ~2857 cm^−1^) were also observed for the double drug-loaded microspheres, and the broadness of the 3377 cm^−1^ peak was attributed to N–H and OH– symmetric stretching vibrations and inter- and intramolecular hydrogen bonds, whereas the weak band at ~2857 cm^−1^ was ascribed to C–H stretching vibrations.

### Surface characteristics of the implant materials

**Figure 2:**
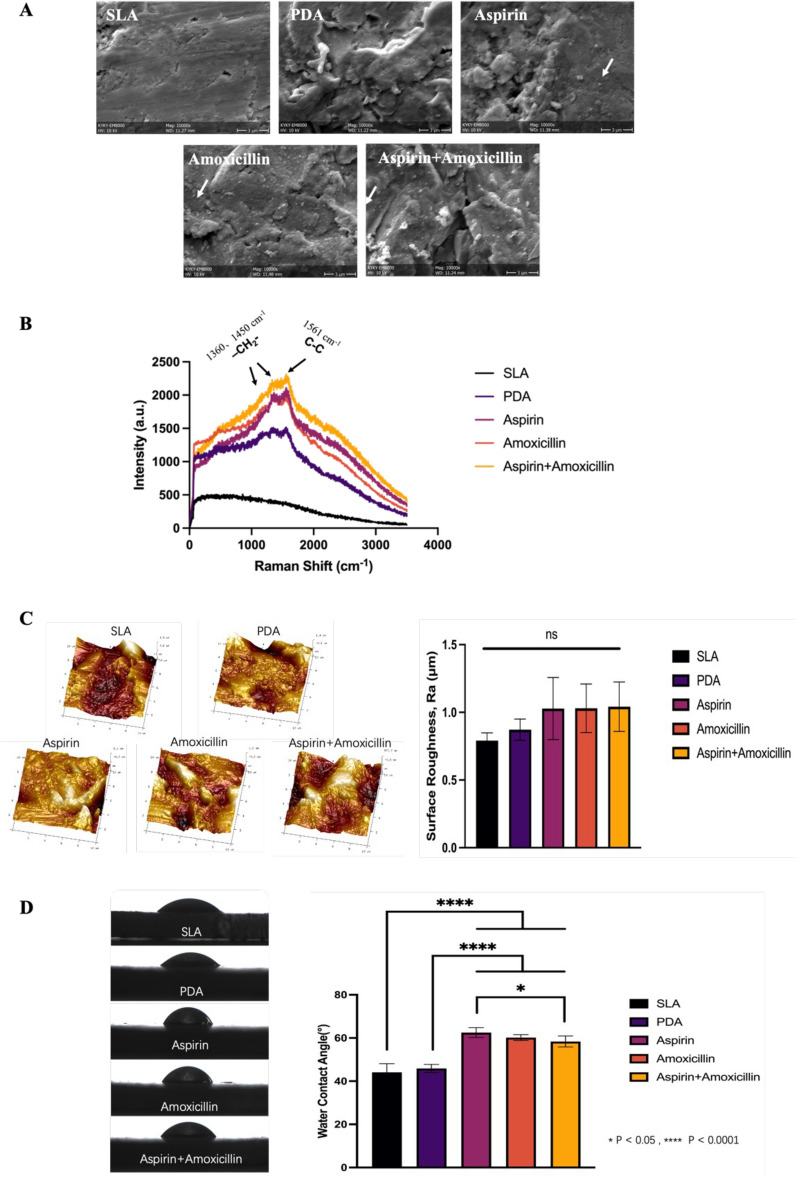
(**A**) SEM images (×10 K) of the modified Ti surface of the SLA group. The PDA group is indicated by a white arrow, and the chitosan particles are indicated by white arrows in the aspirin group, amoxicillin group and aspirin + amoxicillin group. (**B**) Raman spectra of all the groups. (**C**) AFM images of modified Ti surfaces of five groups: SLA surface, PDA surface, aspirin surface, amoxicillin surface and aspirin + amoxicillin surface. There was no significant difference in surface roughness between the five groups (mean ± sd; n = 6). (**D**) Water contact angle of the samples: Representative images of water droplets on the samples and the statistical analysis of the water contact angle (mean ± sd; n = 6) *p < 0.05, ****p < 0.0001.

Different from the smooth Ti surface, uneven marks can be seen on the Ti-SLA surface after sandblasting and acid etching (Fig. 2A). Changes in a relatively smooth Ti surfaces were observed with layered accumulations of polydopamine. White arrows in Fig. 2A indicated the existence of drug-loaded microspheres, and the particles of double drug-loaded microspheres on the surface of the coating.

Qualitative observations of topography on control and experimental Ti surfaces by AFM (Fig. 2C) showed no differences between groups. In addition, no statistical difference in Ra was observed between the different groups tested (p > 0.05).

Using water to assess hydrophilicity, the control SLA group was observed to had a contact angle of 44.12 ± 1.76° (Fig. 2D). Although no significant statistical difference between the PDA and the control groups, an increase in contact angles with aspirin-loaded microspheres (62.52 ± 18.4°), amoxicillin-loaded microspheres (60.19 ± 16.06°), and aspirin + amoxicillin-loaded microspheres (58.37 ± 14.25°). In addition, as observed in Fig. 2B, Raman spectroscopy indicated the existence of –CH_2_-(~1360 cm^−1^, ~1450 cm^−1^) and C–C (~1561 cm^−1^) bonds in microspheres double loaded with amoxicillin and aspirin.

### Adhesion and viability of rBMSCs on Ti surfaces

**Figure 3:**
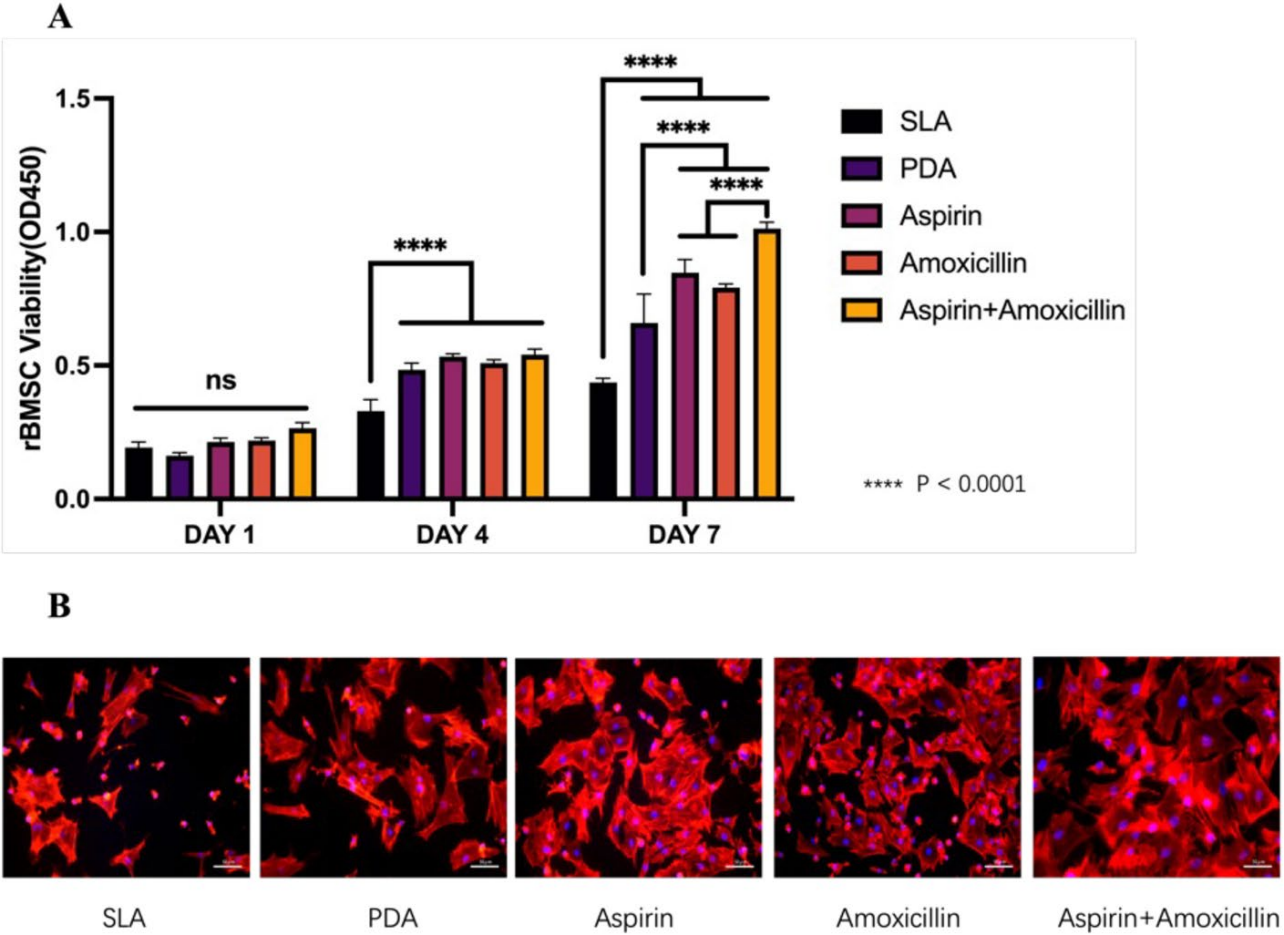
Stem cell adhesion and proliferation on Ti surfaces: (**A**) CCK-8 assay showing the proliferation of rat bone marrow-derived stem cells (rBMSCs) cultured on control and modified surfaces for 1, 4 and 7 days (mean ± sd; n = 6) ****p < 0.0001; (**B**) Cytoskeleton staining of rBMSCs after culture on control and modified surfaces for 3 days (scale bar: 50 μm).

Cell adhesion and proliferation measurements indicated a trend toward increasing cell-promoting effects with increasing coating treatment. A CCK-8 test (Fig. 3A) revealed differences in metabolism beginning at 4 days, and the metabolic level of the cells (p < 0.0001) increased on all the coated Ti surfaces. On Day 7, the cells in the drug-coated groups had higher metabolic levels than did those in the control group and the PDA group (p < 0.0001). Among the three drug-loaded groups, the cells in the aspirin + amoxicillin-loaded microsphere group had a significantly greater metabolic level (p < 0.0001). Figure 3B shows less spreading of cells on the surfaces of the control Ti surfaces, whereas more cell stretching was observed when the cells were exposed to aspirin + amoxicillin-loaded microspheres.

### In vitro evaluation of antibacterial effects

**Figure 4:**
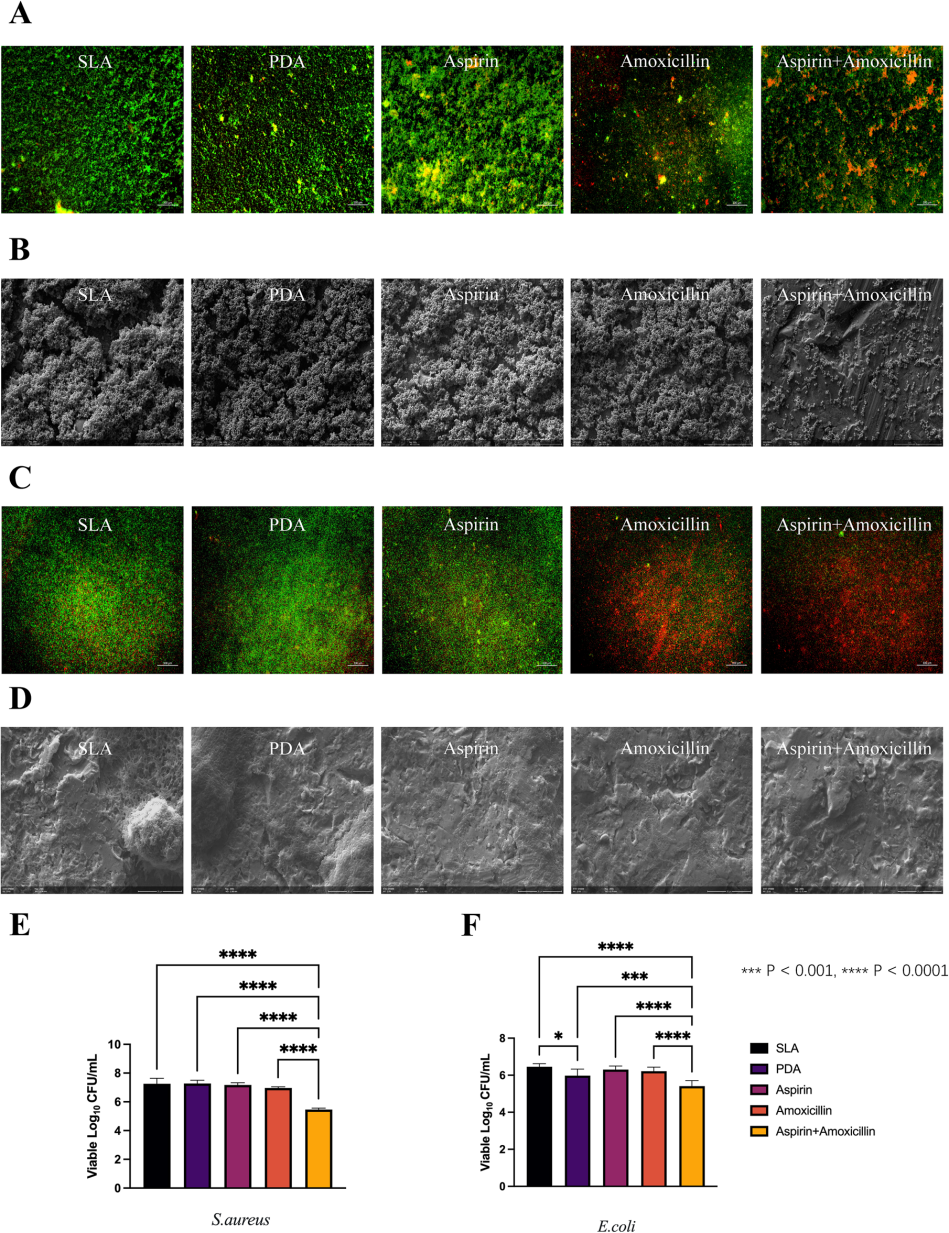
Antibacterial effectiveness of the control and modified surfaces: (**A**) live/dead microscopy images of *S. aureus* adhesion and colonization on different samples (scale bar: 200 μm); (**B**) SEM images (×2 K) of the modified Ti surface cocultured with *S. aureus* biofilm; (**C**) live/dead microscopy images of *E. coli* adhesion and colonization on different samples (scale bar: 100 μm); (**D**) SEM images (×5 K) of the modified Ti surface cocultured with *E. coli* biofilm; (**E**) Statistical analysis results of the *S. aureus* colony count on the surface of titanium tablets in each group (mean ± sd; n = 6) ****p < 0.0001; (**F**) Statistical analysis results of the *E. coli* colony count on the surface of the titanium tablets in each group (mean ± sd; n = 6) ***p < 0.001, ****p < 0.0001.

As shown in Fig. 4A–D, which shows live/dead bacterial staining and SEM images of *S. aureus* and *E. coli* biofilms, fewer viable bacteria were present on surfaces that were exposed to amoxicillin-loaded and aspirin + amoxicillin-loaded microspheres. According to the results of colony-forming unit counting (Fig. 4E), *S. aureus* biofilms that formed on surfaces exposed to the aspirin + amoxicillin-loaded microspheres were also observed to have significantly greater metabolic activity than those in the other groups (p < 0.0001). Like *S. aureus* biofilms, *E. coli* biofilms on surfaces were observed to have significantly lower metabolic activity when exposed to aspirin + amoxicillin-loaded microspheres than when exposed to the other groups tested (Fig. 4F) (p < 0.0001).

### Evaluation of the antibacterial effect in vivo

**Figure 5:**
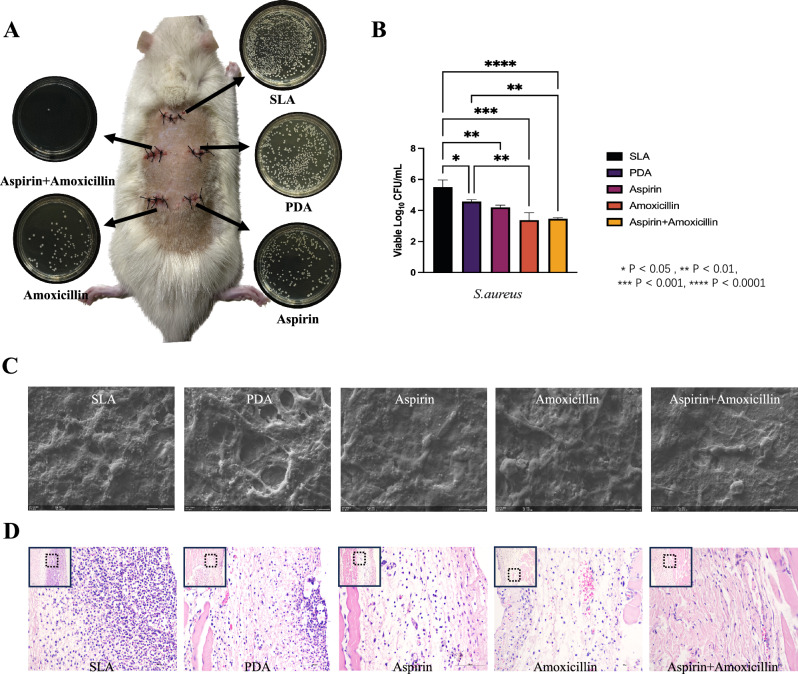
(**A**) Diagram of Sprague–Dawley (SD) rats 24 h after implant surgery. Five respective plate colony counting images are provided to show the amounts of bacteria on the surfaces of the implants. (**B**) Statistical analysis of the *S. aureus* colony counts on the surface of the titanium tablets in each group (mean ± sd; n = 6) ****p < 0.0001. (**C**) SEM images (×3 K) of the modified Ti surface cocultured with *S. aureus* biofilms. (**D**) HE-stained (×20) skin tissues around each group of samples after being cocultured with *S. aureus* in Sprague‒Dawley (SD) rats for 24 h.

In the in vivo antibacterial experiment, the aspirin + amoxicillin-loaded microsphere coating still had a better antibacterial effect. Figure 5A shows that after 24 h of in vivo culture, the number of *S. aureus* colonies on the surface of the titanium tablets decreased significantly in the Aspirin + Amoxicillin group. After statistical analysis of the counts (Fig. 5B), we found that there was no significant difference between the PDA group and the SLA group; all the coatings with drugs had antibacterial effects, but there was no significant difference between the groups with drugs. However, upon observing the surface of the titanium sheet by SEM (Fig. 5C), it could be seen that there were mucinous substances on the surface of the titanium sheet; *S. aureus* adhered to the surface, and the number of *S. aureus* in the Aspirin + Amoxicillin group was significantly reduced. For histopathological analysis, the skin tissues surrounding the implant materials were subjected to HE staining. The tissues around the Ti-SLA implant, which were infected with *S. aureus*, exhibited more strongly inflamed nuclei (Fig. 5D). In contrast, the tissues around the samples in the drug-loaded group that received the same treatment exhibited fewer inflamed nuclei in the skin tissue. The inflammation of the tissues exposed to aspirin + amoxicillin-loaded microspheres was the least severe.

## Discussion

Implant failures are reportedly attributed to a lack of osseointegration and bio-integration as well as the presence of localized infections as a result of bacterial invasion^36^. With the increasing use of implants in the ageing population and longer life expectancy, there is a great need for novel implants that can simultaneously promote or optimize osseointegration, resist or minimize biofilm formation, and maximize implant longevity and success^37^.

In this study, chitosan microspheres were synthesized by a modified double emulsion crosslinking method according to previous methods^35^. Aspirin, amoxicillin, or aspirin + amxicillin was then loaded onto the microspheres, which were subsequently coated on PDA surfaces. As shown in Table 1 and Fig. 1A, the diameter of the microspheres increased when the microspheres were loaded with aspirin + amoxicillin, and the diameter of the aspirin-loaded microspheres was greater than that of the amoxicillin-loaded microspheres. When we synthesized the microspheres, the dosage of aspirin was greater than that of amoxicillin, and the PDI showed that the molecular weight distribution of the aspirin microspheres was wider than that of amoxicillin. The same amount of chitosan coated with more aspirin will form larger microspheres with larger diameter changes. Therefore, the average diameter of aspirin-loaded microspheres will be greater than that of amoxicillin-loaded microspheres. The encapsulation efficiency of each drug was lower (Table 2) in the aspirin + amoxicillin-loaded microspheres than in the aspirin-loaded or amoxicillin-loaded microspheres. The decreased encapsulation efficiency of aspirin + amoxicillin-loaded microspheres may be attributed to competition between the two drugs.

The burst release effect of aspirin^25,38^ has been reported, and it was suggested that small aspirin molecules could easily penetrate through chitosan microspheres. Amoxicillin (molecular mass: 365.4) has a greater molecular mass than aspirin (molecular mass: 180.16), and lighter molecules move at a faster average speed than heavier molecules. We discovered that aspirin was released faster in single aspirin-loaded microspheres and that amoxicillin was released faster in aspirin + amoxicillin-loaded microspheres (Fig. 1C and D). A possible reason for this finding may be that chitosan microspheres have pores^39^, and under the premise of the same chitosan dosage, the larger diameter of double drug-loaded microspheres will have larger pores, which would lead to the faster release of amoxicillin; however, the presence of amoxicillin will compete with aspirin for release from the pores, which would slow the release rate of aspirin.

Coating the control SLA surfaces with PDA or with drug-loaded microspheres did not affect the surface roughness. Using AFM, the roughness of the control and modified Ti surfaces used in this study was observed to be in the range from 0.5 μm to 1 μm, and this range was also reported to be suitable for oral dental implants^40–42^. Using water for contact angle measurements, the hydrophilic surfaces observed for the PDA-coated and drug-loaded microsphere groups were in agreement with previous studies^4,43–46^.

From the rBMSCs studies, CCK-8 test showed higher metabolic level when compared to control SLA and PDA groups (p < 0.0001) on Day 7, and among the three drug-loaded groups, cells showed a significantly higher metabolic level when exposed to aspirin + amoxicillin-loaded microspheres (p < 0.0001), suggesting no cytotoxicity observed. Figure 3B also shows less spreading of cells on the surfaces of the control Ti surfaces, whereas cell stretching was observed when the cells were exposed to aspirin + amoxicillin-loaded microspheres. Another study reported that aspirin-related materials^22,38^ promoted the proliferation of bone marrow mesenchymal stem cells. Studies have shown that the combination of aspirin and other drugs, such as abaloparatide and berberine, has superimposed effects on osteogenic differentiation^47,48^. One study showed that amoxicillin can slow the intestinal flora-mediated consumption of aspirin in the environment, thus prolonging the effect of aspirin^49^. In our study, the aspirin + amoxicillin-loaded group exhibited proliferation and promotion of rBMSCs. The combination of amoxicillin and aspirin may prolong the duration of action of aspirin, thus enhancing osteogenesis. Moreover, aspirin or amoxicillin alone also had an osteogenic effect on cells, and the combined application of aspirin and amoxicillin may have a synergistic effect on osteogenic differentiation. However, further related research is needed.

In addition to the rate of osseointegration, implant success is also strongly dependent on the ability to control microbial colonization, infection and biofilm formation^7^. Amoxicillin is used as a broad-spectrum antibacterial agent in coating antibacterial tests^31^]. In the era where more antibiotics have been prescribed as newer antibiotics are developed, the optimization of antibacterial treatment and increase in antibiotic resistance need to be considered^50^. As shown in the present study, *S. aureus* and *E. coli* exposed to aspirin + amoxicillin-loaded microspheres exhibited significantly decreased metabolic activity. Other investigators^50,51^ have reported the ability of aspirin to enhance the antibacterial effects of antibiotics, and the combined application of aspirin and amoxicillin was reported to alter the morphology of *H. pylori*.

Based on the excellent antibacterial effect of the aspirin + amoxicillin-loaded microsphere surface in vitro, we carried out an animal experiment in vivo. Twenty-four hours after implantation, the SEM images of each Ti disk showed that *S. aureus* was obviously less adherent in the aspirin + amoxicillin-loaded group. From these results, we can see that the antibacterial activity in vivo is consistent with that in vitro. The colony counts showed that the number of colonies in the drug-loaded group decreased significantly from that in the control group. There was no significant difference between the drug-loaded groups, but the difference between the aspirin + amoxicillin group and the control group was even greater (p < 0.001). Because the osteogenic effect can be effectively increased and the number of bacteria can be effectively reduced in the aspirin + amoxicillin group, this coating can be used as a new option for implant surface modification.

## Conclusion

It was concluded from this study that aspirin, amoxicillin, and aspirin + amoxicillin could be loaded into chitosan microspheres using a double emulsion crosslinking method. Although the percent encapsulation efficiency and release of aspirin and amoxicillin are different, the metabolic activity of the rBMSCs exposed to aspirin + amoxicillin-loaded microspheres was significantly greater than that of the rBMSCs in the control SLA groups, whereas the metabolic activity was significantly lower for the *S. aureus* and *E. coli* exposed to aspirin + amoxicillin-loaded microspheres. Moreover, the aspirin + amoxicillin-loaded microsphere coating had good antibacterial effects on rats. It was also concluded that aspirin and amoxicillin could be used in combination to coat implant surfaces to affect cell proliferation and mitigate bacterial activities.

## Data Availability

The datasets generated and/or analysed during the current study are not publicly available due the datasets also form part of an ongoing study but are available from the corresponding author on reasonable request.

## Abbreviations

Ti: Titanium
rBMSCs: Rat bone marrow-derived stem cells
*S. aureus*: *Staphylococcus aureus*
*E. coli*: *Escherichia coli*
ASA: Aspirin
AMO: Amoxicillin
FT-IR: Fourier Transform Infrared
ATR: Attenuated total reflection
SLA: Sandblasted and acid etched
PDA: Polydopamine
AFM: Atomic force microscope
OD: Optical absorbance
PBS: Phosphate buffered saline
LB: Luria–Bertani broth
SEM: Scanning electron microscope
TEM: Transmission electron microscopy
DLS: Dynamic light scattering
CFUs: Colony forming units
SD: Sprague–Dawley
HE: Hematoxylin and eosin
sd: Standard deviations
ANOVA: One-way analysis of variance
EE: Encapsulation efficiency
CS-MPs: Chitosan microparticles
